# The development of an RDoC-based treatment program for adolescent depression: “Training for Awareness, Resilience, and Action” (TARA)

**DOI:** 10.3389/fnhum.2014.00630

**Published:** 2014-08-19

**Authors:** Eva Henje Blom, Larissa G. Duncan, Tiffany C. Ho, Colm G. Connolly, Kaja Z. LeWinn, Margaret Chesney, Frederick M. Hecht, Tony T. Yang

**Affiliations:** ^1^Department of Clinical Neuroscience, Karolinska InstitutetStockholm, Sweden; ^2^Department of Psychiatry, Division of Child and Adolescent Psychiatry, University of California San FranciscoSan Francisco, CA, USA; ^3^Department of Family and Community Medicine, University of California San FranciscoSan Francisco, CA, USA; ^4^Osher Center for Integrative Medicine, University of California San FranciscoSan Francisco, CA, USA; ^5^Department of Medicine, University of California San FranciscoSan Francisco, CA, USA

**Keywords:** adolescent depression, RDoC, treatment development, emotion regulation, attention training, yoga-based movement, mindfulness

## Abstract

Major depressive disorder (MDD) is one of the current leading causes of disability worldwide. Adolescence is a vulnerable period for the onset of depression, with MDD affecting 8–20% of all youth. Traditional treatment methods have not been sufficiently effective to slow the increasing prevalence of adolescent depression. We therefore propose a new model for the treatment of adolescent depression *– Training for Awareness, Resilience, and Action (TARA) –* that is based on current understanding of developmental and depression neurobiology. The TARA model is aligned with the Research Domain Criteria (RDoC) of the National Institute of Mental Health. In this article, we first address the relevance of RDoC to adolescent depression. Second, we identify the major RDoC domains of function involved in adolescent depression and organize them in a way that gives priority to domains thought to be driving the psychopathology. Third, we select therapeutic training strategies for TARA based on current scientific evidence of efficacy for the prioritized domains of function in a manner that maximizes time, resources, and feasibility. The TARA model takes into consideration the developmental limitation in top-down cognitive control in adolescence and promotes bottom-up strategies such as vagal afference to decrease limbic hyperactivation and its secondary effects. The program has been informed by mindfulness-based therapy and yoga, as well as modern psychotherapeutic techniques. The treatment program is semi-manualized, progressive, and applied in a module-based approach designed for a group setting that is to be conducted one session per week for 12 weeks. We hope that this work may form the basis for a novel and more effective treatment strategy for adolescent depression, as well as broaden the discussion on how to address this challenge.

## INTRODUCTION

The World Health Organization (WHO) identifies major depressive disorder (MDD) as one of the current leading causes of disability worldwide (Ferrari et al., 2013). Adolescence is a vulnerable period for the onset of depression, with MDD affecting 8–20% of all youth (Kessler et al., 2007; Thapar et al., 2012). MDD is often recurrent and early onset MDD predicts a fourfold increase in the risk of developing adult depression (Naicker et al., 2013). Depressed adolescents are at higher risk of future psychiatric morbidity including substance use disorder, cognitive impairment, and increased risk of suicide (Birmaher et al., 2002). Approximately 13–20% of the children living in the United States experience a psychiatric disorder often including depressive disorders, with suicide being the second leading cause of death among US children aged 12–17 years in 2010 (Perou et al., 2013). During 1994–2011, the prevalence of psychiatric conditions increased, and is now estimated to cost the U.S. $247 billion annually (Perou et al., 2013). Thus, early detection and effective treatment methods would relieve the burden imposed by MDD on the aﬄicted individual and society as a whole.

Traditional treatment methods for adolescent depression such as anti-depressive medication and psychological cognitive strategies such as cognitive behavioral therapy (CBT) have not been sufficiently effective to slow the increasing prevalence of this disorder. Follow-up studies of both pharmacotherapy and psychotherapy randomized controlled trials (RCTs) show that 25–50% of the depressed adolescents relapse within 6 months to 2 years post-treatment (March and Vitiello, 2009). A recent Cochrane meta-analysis concluded that there is very limited evidence demonstrating the relative effectiveness of antidepressant medication, psychological interventions, and a combination of these interventions in depressed youth (Cox et al., 2012). However, despite the fact that pharmacological treatments of mild to moderate adolescent MDD have not shown significant treatment effects and may introduce both short- and potential long-term negative side effects (Hetrick et al., 2007; Adegbite-Adeniyi et al., 2012), 14.1% of adolescents with primary mood disorders are treated with antidepressant medication in the U.S. (Merikangas et al., 2013).

Thus far, the quest for biomarkers that may better characterize specific subtypes of depression or aid in the personalization of depression treatment has not yet improved treatment efficacy for adolescent depression. Furthermore, the advances in scientific understanding of neuroplasticity and neurodevelopment have not been translated into clinical applications or, have yielded established treatment models of adolescent depression. We therefore propose a new model for the treatment of adolescent depression *– Training for Awareness, Resilience, and Action (TARA) –* that is based on our current understanding of developmental and depression neurobiology. In designing the TARA program, we have carefully considered the key elements of neural plasticity that were highlighted in the National Institute of Health Blueprint for Neuroscience Research Workshop for creating successful clinical interventions: time-sensitivity and the creation of a platform for attention and behavioral motivation (Cramer et al., 2011). The TARA program for adolescent depression is time sensitive since we intervene during peak brain plasticity. We propose to use breathing exercises, yoga-based movement and short meditation practices to create the foundation for improved attention. The motivation for behavioral change in the TARA program is linked to subjective experience and guided by the participant’s own core values. The intervention is designed in a progressive and module-based manner so that the mechanisms that drive the psychopathology are first addressed.

In this paper, we first introduce the Research Domain Criteria (RDoC) and its relevance to the research field of adolescent depression. We then describe the domains of function central to the psychopathology of adolescent depression and organize these domains of function hierarchically, prioritizing the domains of function that are thought to be driving the psychopathology. Next, we selected therapeutic and training strategies based on their feasibility and the current scientific evidence to target these domains of function. We outline our selection process in a pragmatic way to fit our neurodevelopmental, neurobiological, and metabolic theory of change. Finally, we present a structured, progressive, and individually adaptable module-based program in which the first step consists of techniques targeting the domain of function highest in the hierarchy.

## ALIGNMENT WITH THE NATIONAL INSTITUTE OF MENTAL HEALTH’S RESEARCH DOMAIN CRITERIA

The National Institute of Mental Health’s (NIMH) RDoC Project, as described by Insel et al. (2010) intends to “develop a research program which incorporates data on pathophysiology in ways that eventually will help identify new targets for treatment development, detect subgroups for treatment selection, and provide a better match between research findings and clinical decision making” (Insel et al., 2010). It is based on the assumption that mental illnesses are related to dysfunction of relevant brain circuits, which can be identified by clinical neuroscience. The levels of analysis progress from measures of circuitry function upward to clinical manifestations or downward to the genetic and molecular/cellular factors influencing these functions. The RDoC project identifies five broad domains of function, which may contribute to psychopathology in varying degrees across a range of clinically defined psychiatric disorders (Insel et al., 2010): negative valence, positive valence, cognition, social processes and arousal/modulation. The structure of the RDoC is described as “a matrix in which the rows represent various constructs grouped hierarchically into broad domains of function. The columns of the matrix denote different levels of analysis, from genetic, molecular, and cellular levels, proceeding to the circuit-level to the level of the individual, family environment, and social context” (Insel et al., 2010). For a graphic illustration of the RDoC matrix, see (http://www.nimh.nih.gov/research-priorities/rdoc/nimh-research-domain-criteria-rdoc.shtml#toc_matrix).

The application of the RDoC approach instead of the Diagnostic and Statistical Manual of Mental Disorders (DSM) system is especially relevant in the research field of adolescent depression. The DSM diagnostic criteria for adolescent MDD have low diagnostic validity and specificity, with unclear diagnostic boundaries (Costello et al., 2003; Ford et al., 2003; Korczak and Goldstein, 2009; Alexandrino-Silva et al., 2012; Henje Blom et al., 2014; Schuch et al., 2014). Psychiatric comorbidity is also highly prevalent in adolescent depression (Angold and Costello, 1993; Middeldorp et al., 2005; Spencer, 2006; Hettema, 2008). These factors contribute to heterogeneous sample configurations when recruiting depressed adolescents for research on the basis of DSM criteria and makes it very hard to tailor an effective treatment for the heterogeneous population that the DSM MDD diagnosis will encompass. Consequently, from a neuroscientific research perspective it may be more relevant to study the different brain circuits implicated in adolescent depression, such as those important to emotion regulation, attention, motivation, stress responses, social cognition, reward processing, and neuro-vegetative functions in patients who may potentially span multiple DSM diagnoses (Joormann and Goodman, 2014). Ultimately, brain development should also be taken into account in the RDoC constructs since the implicated brain circuits mature within different time frames and thus give rise to age-dependent symptomatology (Drevets et al., 2008). The neurocircuitry implied in adolescent depression often overlaps between different domains and constructs. Therefore, to maximize time, resources, and feasibility in designing an intervention model for adolescent depression, we suggest specific neural circuits to be targeted in our treatment model rather than all of the neurocircuitry described in each construct. While a RDoC based approach to develop a treatment program for geriatric depression has previously been developed and applied (Alexopoulos and Arean, 2014), our approach to use the RDoC in combination with developmental aspects of the brain as the foundation for creating a treatment model for adolescent depression is the first of its kind.

The transition from the DSM system to RDoC is a challenge in our research design, since on the one hand we have adolescent depression as a starting point, which is a diagnostic entity (i.e., MDD) that is constituted of symptoms that may arise from different RDoC constructs and originate in different neural pathways, and on the other hand we try to tease out distinct neurocircuitry that have a behavior or symptom-related feature which is possible to identify across different DSM diagnostic entities. Our solution to this challenge is a pragmatic one: to base our hypothesis on the most common clinical manifestations of adolescent depression, which is not congruent with the MDD DSM categories and which extend beyond one specified RDoC construct. Consequently, both unspecific and specific symptoms according to the DSM will be addressed.

In summary, the RDoC approach gives us an opportunity to address specific dimensions of psychopathology in a hierarchical manner according to what we hypothesize drives the pathophysiology of adolescent depression. For instance, giving priority to target anxious arousal, worrying and rumination specifically, allows us to assess these dimensions from a neuroscience perspective. We aim to find out whether this approach will aid the development of a more targeted and efficient treatment for adolescent depressive symptoms.

## BRAIN DEVELOPMENT AND VULNERABILITY TO DEPRESSION

During childhood, the brain undergoes an overproduction of synapses and gray matter. Throughout normal brain development, excess neural connections are then progressively pruned (Giorgio et al., 2010). Subcortical areas, such as the brainstem and limbic structures involved in basic functions and emotional processing mature earlier in life (Gogtay et al., 2004). Higher order cortical regions, specifically the prefrontal and frontal cortices responsible for executive functioning and cognitive control are not fully developed until the mid-20’s (Giorgio et al., 2010; Fjell et al., 2012). Thus, the adolescent brain differs from the adult brain in frontal development and frontolimbic connections (Gogtay et al., 2004). It has been suggested that altered development of these neural circuits increases the risk for depression (Andersen and Teicher, 2008; Pandey et al., 2013). The differing rates between subcortical and cortical maturation during adolescence may increase vulnerability to emotional reactivity, as the absence of effective top-down regulation from prefrontal and frontal regions on limbic reactivity may be a possible contributing mechanism to developing depressive symptoms (Cunningham et al., 2002; Gogtay et al., 2004; Yurgelun-Todd and Killgore, 2006). This neural pattern of development that subserves limited top-down control may translate into behavioral patterns such as impulsivity, risk-taking, novelty-seeking, increased emotional intensity and reactivity in adolescents (Gogtay et al., 2004; Giorgio et al., 2010; Tamura et al., 2012; Rubia, 2013). All of these factors may be functionally adaptive during this developmental period as increased peer-group social focus and orientation toward autonomy is essential in adolescence. However, these factors may simultaneously constitute an increased vulnerability to depressive symptomatology (Ernst and Korelitz, 2009). To alter negative trajectories leading to depressive illness and to avoid the lifelong recurrent pattern of depression, we should intervene with effective prevention and treatment in childhood and adolescence when neuronal plasticity is at its peak and before secondary co-morbidities occur (Hulvershorn et al., 2011).

## THE NEUROCIRCUITRY CENTRAL TO THE PSYCHOPATHOLOGY OF ADOLESCENT DEPRESSION

To date, the body of literature on adolescent depression using functional magnetic resonance imaging (fMRI) has shown consistent alterations in the amygdala, the anterior cingulate cortex (ACC), in particular the subgenual portion (sgACC) and the medial prefrontal cortex (MPFC). The amygdala is a limbic structure that plays a key role in emotion-processing and memory formation (Morris et al., 1998; LeDoux, 2007). The sgACC acts as an interface between emotion and cognition with evidence of involvement in both emotion generative and regulatory processing (Seminowicz et al., 2004). The MPFC is implicated in many functions, including emotion regulation, reward processing, memory consolidation, and self-referential processing (Buckner et al., 2008). Both adolescent (Buckner et al., 2008; Yang et al., 2010; Perlman et al., 2012) and adult MDD (Sheline et al., 2001; Cunningham et al., 2002) are associated with amygdalar hyperactivation in response to emotional stimuli, and increased amygdalar activation has been shown to correlate positively with depression severity (Price and Drevets, 2010; Lane et al., 2013). The amygdala has reciprocal connections with the sgACC, which has also been demonstrated to exhibit resting-state hyperperfusion (Ho et al., 2013) and task-related hyperactivation in depressed compared to healthy adolescents (Yang et al., 2009). More recently, functional connectivity (FC) of the sgACC during resting-state (Cullen et al., 2009; Davey et al., 2012a; Connolly et al., 2013) and during emotional processing (Ho et al., 2014) has been assessed to investigate the functioning of neural networks in adolescent depression. Two of these studies showed increased sgACC-amygdala FC in depressed compared to healthy adolescents (Connolly et al., 2013; Ho et al., 2014). It is hypothesized that enhanced bottom-up responses to affectively laden stimuli could be linked to deficits in amygdala and sgACC function and represent heightened emotional reactivity to emotional and social stimuli in adolescent MDD. Similar results have been found in MDD patients in remission (Joormann and Gotlib, 2007; Fritzsche et al., 2010) and in high-risk girls (Joormann et al., 2012), thereby suggesting that limbic and cingulate hyperactivation may be a trait which increases vulnerability to depression. These bottom-up processes could potentially also have negative effects on the development of cognitive control mechanisms involved in physiologic and behavioral coping by sustaining negative affect (Fales et al., 2008; Michl et al., 2013). Depressed individuals often ruminate and have difficulties disengaging from the processing of self-focused, often negatively biased thoughts (Cooney et al., 2010; Manoliu et al., 2013; Mandell et al., 2014). It has been proposed that aberrant switching from self-referential processing involving midline prefrontal structures such as the MPFC to interoceptive processes involving the insula or to goal-directed cognitive processes involving the dorsolateral prefrontal cortex (DLPFC) might contribute to MDD (Manoliu et al., 2013).

The insula is a paralimbic structure implicated in a broad range of different functions (Craig, 2011; Nieuwenhuys, 2012). A dysfunction in the integration of visceral signals to the insular cortex has been suggested in adult depression (Critchley, 2005) and adult MDD is associated with abnormal functional connectivity of the insula (Mutschler et al., 2012; Avery et al., 2013). Interestingly, increased sgACC-insula functional connectivity (Connolly et al., 2013) has been documented in adolescent depression during resting-state, whereas sgACC-insula functional connectivity during emotion processing in this population has been shown to decrease (Ho et al., 2014). This may indicate that altered sgACC-insula connectivity does characterize adolescent MDD, but that the directionality of the functional connections change with brain state (Ho et al., 2014).

Cognitive control areas within portions of the PFC, such as the DLPFC and the ventrolateral prefrontal cortex (VLPFC) have been implicated in adult MDD (Rive et al., 2013). It has been suggested that reduced DLPFC recruitment in depressed adults is present only when the amygdala is overactive, indicating that dysfunction in the DLPFC may not the primary mechanism involved in adult MDD but that amygdalar hyperactivity may have a bottom-up influence on the level of activity in DLPFC (Drevets et al., 2008). The DLPFC does not seem to be as strongly implicated in adolescent as compared to adult MDD (Kerestes et al., 2013). This is in line with the lack of evidence for differences between depressed and healthy control adolescents in both behavioral performance and brain activation in DLPFC during tasks of cognitive control (Davey et al., 2012b; Bora et al., 2013). In fact, cognitive deficits in MDD seem to become more severe with recurrent MDD and are more pronounced in late-life depression (Koenig and Butters, 2014). It must be emphasized that adolescents in general, independent of depressive illness, have functional limitations in top-down control systems compared to adults due to differing frontal development and frontolimbic connections (Gogtay et al., 2004; Giorgio et al., 2010; Tamura et al., 2012; Rubia, 2013). Task-dependent DLPFC activation in adolescents has been shown to measures of impulse control, foresight, and resistance to peer pressure (Andrews-Hanna et al., 2011). The limitations in executive function and cognitive control may thus contribute to the increased risk of depression and certain aspects of adolescent depression symptomatology, but we suggest that these mechanisms are not primarily driving the pathophysiology of adolescent MDD.

It should be mentioned that the dysfunction of specific brain regions described in adolescent depression should not be regarded in isolation, but rather as part of networks of structures functionally interacting together (Greicius, 2008; Hamilton et al., 2013). Studies of adult MDD suggest dysfunction in an extended medial network involved in emotion processing (Price and Drevets, 2012). This network partly overlaps with and is dynamically interacting with the DMN, which is involved in self-referential processing (Buckner et al., 2008; Greicius, 2008; Hamilton et al., 2013). The extended medial network is also functionally interacting with the executive control network implicated in cognitive control of emotions (Phan et al., 2004). Similar network dysfunctions has been partly suggested in adolescent MDD (Kerestes et al., 2013).

Alterations in reward circuitry have also been implicated in the pathophysiology of adolescent MDD (Forbes and Dahl, 2012). Functional MRI studies using reward processing tasks show decreased activation in the striatum and increased activation in MPFC in response to reward in depressed adolescents compared to healthy controls (Forbes and Dahl, 2012). In addition, adolescents with MDD show increased resting-state FC between striatal regions and midline structures (Gabbay et al., 2013). Furthermore, alterations in striatal FC correlate with depression and anhedonic severity (Gabbay et al., 2013). Dysfunctional social reward systems may be particularly implicated in adolescent depression, since depression is often precipitated by social rejection or loss of status in this age-group (Davey et al., 2008). Altered reward circuitry may also be specifically related to anhedonia, which is prevalent but variable among depressed adolescents (Gabbay et al., 2009, 2010, 2012; Geisler et al., 2013). Converging evidence suggests that anhedonia specifically reflects disturbances in a mesolimbic striatum-based reward system in adolescents with MDD, whereas MDD without anhedonia is accompanied by normal function in this circuitry (Gabbay et al., 2013). Anhedonia in adolescence predicts chronicity of MDD (Bennik et al., 2013), is a negative prognostic predictor for treatment response (McMakin et al., 2012), and MDD with anhedonia may constitute more severe type of adolescent depression (Gabbay et al., 2013). The anhedonic form of adolescent depression has a symptom profile similar to adult melancholic depression (Gabbay et al., 2013). In contrast, the adolescent form of depression without anhedonia but with emotion instability/dysregulation, stress reactivity, low impulse control and comorbid anxiety shows a dramatic increase of prevalence at the onset of puberty with a major predominance in girls. This gender effect is hypothesized to be related to estrogen levels and cortisol receptor sensitivity in the brain (Antonijevic, 2006; Paing et al., 2008; Weiser and Handa, 2009; Smith, 2012). The non-anhedonic symptom profile in adolescent depression has similarities with the features of atypical depression in adults according to the DSM system, even though the diagnostic validity is low for atypical depression according to DSM in this age-group (Williamson et al., 2000; Paing et al., 2008). Notably, women have a documented higher frequency of comorbid depression and anxiety disorders, and a threefold higher prevalence of atypical depression compared to men (Silverstein, 2002; Halbreich and Kahn, 2007). The female predominance of depression in adults thus seems to be restricted to the atypical subtype in premenopausal women (Angst et al., 2002; Silverstein, 2002). This contrasts with the pattern seen in postmenopausal women, for whom melancholic MDD is predominant and no gender differences have been identified (Antonijevic, 2006). The literature describes differences in hypothalamic-pituitary-adrenal (HPA) axis function, serotonin and noradrenaline function, immune system activation and treatment response between MDD and atypical depression (Angst et al., 2006; Antonijevic, 2008). Based on this evidence, we hypothesize that the pathophysiological mechanisms involved in atypical depression in women are similar to those in the most common form of depression in adolescent females.

In mid-puberty a 2:1 female to male ratio in depressive disorders emerges (Angold et al., 1998). A large body of literature suggests that an estrogen dependent increase of stress sensitivity and mood instability increases the vulnerability to depression in puberty for girls (Angold et al., 1998, 1999; Steiner et al., 2003; Ter Horst et al., 2009; Weiser and Handa, 2009; Blanton et al., 2012; Smith, 2012).

Summarizing these findings, two possible pathophysiological trajectories leading to adolescent depression emerge. One subtype seems to be driven by *limbic hyperactivation*, related to sustained threat with clinical features such as emotional hyper-reactivity, agitation and dysphoric mood and possible secondary negative effects on the ability to dynamically switch from self-referential processing to interoceptive awareness or goal directed behavior. A second subtype may be driven by *compromised positive reward circuitry* and may be related to an anhedonic subtype of adolescent depression (Gabbay et al., 2013). Based on clinical experience and the literature of atypical versus melancholic depression according to the DSM system (Antonijevic, 2006; Paing et al., 2008), we suggest that the increased prevalence of MDD seen in puberty is mainly constituted by a subtype that best fits the symptomatology of limbic hyperactivation corresponding to the RDoC construct of “sustained threat.” The subtype of adolescent MDD that may be more related to dysfunction of reward circuitry seems to remain more stable across different age groups and gender (Antonijevic, 2006; Halbreich and Kahn, 2007). When creating the foundation for an intervention for adolescent depression by determining the hierarchy of the domains of function, it is crucial to identify which of these subgroups to target. In our present work, we chose to focus on the most prevalent type of adolescent depression with clinical features of emotional hyper-reactivity, agitation, and dysphoric mood but without major signs of anhedonia. In the future our manual can be adapted for other types of depression as well.

## THE ROLE OF SUSTAINED THREAT IN RELATION TO SYSTEMIC DYSREGULATION AND THE SOMATIC MANIFESTATIONS OF DEPRESSION

The PFC and the amygdala are linked via the vagus nerve to the regulation of metabolic systems (Thayer and Sternberg, 2006). It is hypothesized that a predisposition to amygdala and limbic hyperactivation and decreased vagal control, such as is found in depressive illness, will induce a cascade of dysregulation across multiple systems (Juster et al., 2010). Inflammatory processes (Henje Blom et al., 2011; Maes et al., 2011), glucose–insulin homeostasis, glucocorticoid signaling, oxidative stress, and energy biosynthesis (Thayer and Sternberg, 2006; McIntyre et al., 2007) are examples of systems that are often dysregulated in depression. Over time, this dysregulation can build to an increased risk of developing systemic diseases such as atherosclerosis, heart disease, hypertension, stroke, osteoporosis, deterioration of the immune system, obesity, metabolic syndrome, insulin resistance and Type 2 diabetes (Wolkowitz et al., 2011).

Early childhood experience may be one pathway through which neural circuits underlying depression may become dysregulated. Childhood maltreatment may cause patterns of limbic hyper-responsiveness and increased rumination (Michl et al., 2013), which may consequently lead to altered and sustained physiological responses to stress, including mechanisms of inflammation, oxidative stress, telomerase activity and regulation of neurotrophic factors, growth factors, and neurosteroids (Taylor et al., 2011; Shonkoff and Garner, 2012; Karatsoreos and McEwen, 2013). The effect of chronic childhood abuse and adverse life events on neurosteroid metabolism during development has been especially well studied and described in the RDoC construct of sustained threat (Heim et al., 2000). The implicated mechanism is HPA dysregulation with persistent corticotropin-releasing factor (CRF) hypersecretion, overproduction of adreno-corticotropic hormone (ACTH) in the anterior pituitary and increased glucocorticoid release from the adrenal glands (Heim et al., 2000). Preclinical studies show that increased glucocorticoid secretion progressively reduces neurogenesis and synaptogenesis in the hippocampus (Andersen and Teicher, 2008). This damage to hippocampal neurons then contributes to hippocampal dysfunction and the eventual development of depression (Heim et al., 2000; Kaymak et al., 2010; Dannlowski et al., 2012; Teicher et al., 2012). Regional differences in synaptic development and the establishment of glucocorticoid receptors may also influence the development of other specific regions and functional connectivity among these regions during adolescence. Early life stress has been shown to increase childhood cortisol levels, which predicts decreased amygdala-ventromedial prefrontal cortex (vmPFC) connectivity in adolescence (Burghy et al., 2012). Therefore, there may be a temporal delay between early stress exposure and the clinical manifestations of depression (Andersen and Teicher, 2008).

Smaller hippocampal volumes have been a consistent finding in depressed adults (Kempton et al., 2011) and in early onset depressed adolescents (Kaymak et al., 2010; Hulvershorn et al., 2011). However, in child- and adolescent depression, hippocampal size is positively correlated with the duration of illness, which is not the case in adult depression (Hulvershorn et al., 2011). This may be a compensatory process in the developing brain aimed at neural recovery (Hulvershorn et al., 2011). This is important from a clinical perspective since it implies that reduction of hippocampal volume may be prevented by effective interventions during the sensitive time-period by which this compensatory mechanism is still at work. This finding is also in line with previous data showing that children who are exposed to maternal depressive symptomatology since birth show increased levels of glucocorticoids but not changes in hippocampal volume (Lupien et al., 2011). On the other hand, these children show increased amygdala volumes, which indicates that the amygdala may be particularly sensitive to early life adversity and stress (Lupien et al., 2011).

Based on these findings, we hypothesize that exposure to sustained threat is detrimental to the developing central nervous system. A negative spiral seems to be created wherein amygdala hyperactivity leads to increased metabolic dysfunction, which in turn instigates dysfunction of emotion regulation circuitry and increased sensitivity to threatening cues, further increasing the risk of metabolic consequences (McIntyre et al., 2007). The trajectory of depressive disease starts early and continues unless addressed by effective and targeted interventions (McEwen, 2003; Juster et al., 2010; Dannlowski et al., 2012). We therefore suggest that the first priority in treating pediatric depression should be to normalize amygdala and limbic hyperactivity and ACC dysfunction by specially designed practice as outlined in the later sections of this article.

## HIERARCHICAL ORGANIZATION OF THE RDoC DOMAINS OF FUNCTION

We have identified the major RDoC domains of function involved in adolescent depression and organized them in a way that gives priority to domains thought to be driving the psychopathology (see **Table 1**). We propose that the most important factor driving the pathophysiology of mild to moderate adolescent depression without major signs of anhedonia is limbic hyper-reactivity and dysregulation of the amygdala and ACC as previously described. These neural circuits correspond well to **the RDoC construct of sustained threat within the domain of negative valence.** The behavioral unit of analysis of the RDoC construct of sustained threat describes several of the core symptoms of the most common type of adolescent depression: anxious arousal, increased conflict detection, attentional bias to threat, helplessness behavior, punishment sensitivity and avoidance. These mechanisms are supported by the extensive body of literature suggesting an increased risk of adolescent depression as a result of childhood trauma (Andersen and Teicher, 2008; Taylor et al., 2011; D’Andrea et al., 2012; Dannlowski et al., 2012; Gulec et al., 2013) and it is also congruent with the findings that anxious symptomatology often precedes the onset of MDD (Stein et al., 2001) and that anxiety disorders are highly comorbid with MDD in this age group (Keller et al., 1992; Angold and Costello, 1993; Seligman and Ollendick, 1998; Costello et al., 2003; Middeldorp et al., 2005; Olino et al., 2008).

**Table 1:** A proposed hierarchy of RDoC constructs relevant in treating mild to moderate adolescent depression without major anhedonia.

RDoC constructs	RDoC domains	Implicated neurocircuitry	Clinical manifestations	Intervention strategies
**Driving depressive pathophysiology**
Sustained threat	Negative valence	Limbic hyper-reactivity with dysregulation of the amygdala and the anterior cingulate cortex	Anxious arousal; increased conflict detection, attentional bias to threat, helplessness behavior, punishment sensitivity, avoidance	Stress-reduction by breathing exercises and yoga-based movement
Arousal and sleep-wakefulness	Arousal and regulatory systems	Hypothalamic to thalamic and cortical circuits	Sleep-wake dysregulation and sleep dependent behavioral dysfunctions such as affect regulation, emotional reactivity, impulsivity	Decrease of daytime hyper- arousal by breathing exercises and yoga-based movement. Psycho-education related to sleep
Loss	Negative valence	Sustained amygdala reactivity and decreased DLPFC recruitment. Increased default mode activity	Sadness, guilt, shame, low self-esteem, worry, rumination, increased self-focus, withdrawal behavior	Practice of dynamic shifting from rumination to interoceptive awareness. Cognitive reappraisal techniques
**Maintaining depressive psychopathology**
Attention and cognitive control	Cognitive systems	Circuitry involving top down cognitive control: DLPFC, VLPFC, PPC, the insula and limbic system	Concentration difficulties, distractibility. Low impulse control	Practice of attention by body-scans and sitting meditation
**Of interest for treatment approach**
Approach motivation	Positive valence	Circuitry involving MPFC, OFC, dorsal and ventral striatum and amygdala		Practice approach behavior rather than experiential avoidance
Reward learning	Positive valence	Circuitry involving OFC and ventral striatum		Core value identification, practicing value-based decision making for behavioral activation
Social communication, perception and understanding self and others and self knowledge	Social processes	Broad range of brain areas, beyond the scope of this table		Practice to care for oneself and others and to receive care/compassion. Practice to communicate emotions. Practice of understanding one-self in relation to others

*Since the implied neurocircuitry often overlaps between different RDoC domains and constructs we suggest that only specific neural circuits are targeted for treatment as suggested in the column “implicated neurocircuitry” rather than all of the neurocircuitry described in each construct.*

Closely related to the sustained threat mechanism is autonomic nervous system hyper-reactivity **and dysfunction in the domain of arousal and regulatory systems both within the RDoC construct of arousal and sleep-wakefulness.** Neurocircuits related to sleep and to arousal are reciprocally modulating each other. This is represented by reciprocal connections from the amygdala to other limbic structures such as the thalamus and hypothalamus, as well as to cortical structures (McGinty and Szymusiak, 2003; Szymusiak et al., 2007). Insomnia may be secondary to frequent daytime hyper-arousal (Riemann et al., 2010) and it is hypothesized that sleep disturbances precede and are etiologically linked to the emergence of depressive symptoms (Harvey et al., 2011). Persistent sleep disturbance may cause secondary cognitive dysfunctions, daytime tiredness, concentration difficulties, affect dysregulation, emotional reactivity, impulsivity and dysregulation of neuroendocrine and immunological systems and it is negatively related to depression treatment outcome (Manglick et al., 2013).

Dysphoric mood, including sadness, guilt, shame, and low self-esteem are core features of adolescent MDD, which correspond to **the construct loss, within the negative valence RDoC domain.** Worry, rumination, increased self-focus, withdrawal behavior, and impaired sustained attention are examples of other common features of adolescent depression described in the construct loss, that may be secondary to sustained threat mechanisms as previously described. However, it is important to emphasize that several of the behavioral features described within the RDoC construct of loss such as psychomotor retardation, anhedonia, loss of drive (sleep, appetite, libido), and a lack of motivation may constitute a distinct and less prevalent subtype of adolescent depression in which dysfunctional reward circuitry may be implicated. As previously described, we acknowledge that an aberrant reward circuitry exists in adolescent depression as shown in previous functional MRI (Forbes et al., 2007, 2009; Forbes and Dahl, 2012) and magnetic resonance spectroscopy studies (Gabbay et al., 2007). We suggest that these findings may be related to a more severe form of anhedonic depression and that aberrant reward circuitry may not be driving the pathophysiology of mild to moderate adolescent depression, in which the previously described mechanisms of a hyper-reactive limbic system are more dominant.

The DSM-IV definition of MDD includes several more general psychiatric symptoms, which may be part of the DSM diagnostic criteria of other psychiatric disorders as well. These unspecific symptoms may still contribute to and maintain the psychophysiology of depression and be part of the suggested RDoC constructs. Concentration difficulties, for example, are often part of adolescent depression symptomatology and belong to **the construct of attention in the domain of cognitive systems** (Han et al., 2012). Another unspecific symptom associated with adolescent behavior in general is impaired impulse control (Andrews-Hanna et al., 2011) which is described as a **within the cognitive control construct in the domain of cognitive systems** in the RDoC matrix as represented by impaired top-down regulation from DLPFC, VLPFC, and posterior parietal cortex (PPC). The decrease of impulse control in combination with dysphoric mood may be driving secondary comorbidities such as behavioral problems, self-harming behavior, drug abuse, and suicidal actions.

Constructs within the **positive valence system** domain may constitute relevant mechanisms to consider when designing a treatment model for adolescent depression. We hypothesize that dysfunction of mechanisms of **approach motivation** may relate to increased experiential avoidance, which may be involved in sustaining depressive symptomatology. The **reward learning construct within the positive valence system** also describes value-based decision-making behavior that may increase resilience to depressive symptomatology by contributing with behavioral motivation, meaning and direction in the life of the adolescent. Finally, dysfunction within the domain of **social processes** such the constructs of **social communication, perception and understanding self and others and self knowledge** is also important in the treatment of adolescent depression since depressive self-evaluation and extensive negative self-referential processing often impede social connections and a healthy peer group identification that is developmentally important in adolescence.

In summary, we propose the following hierarchy of RDoC constructs in treating mild to moderate adolescent depression without major anhedonia (see also **Table 1**):

**1. Sustained threat** with symptoms of anxious arousal, increased conflict detection, attentional bias to threat and emotion-laden stimuli, helplessness behavior, punishment sensitivity and avoidance.
**2. Arousal and wakefulness** including limbic and autonomic hyperarousal with manifestations such as insomnia and secondary sleep dependent behavioral dysfunctions.
**3. Loss**, including feelings of sadness, guilt, shame, low self-esteem, worry, rumination, increased self-focus, and withdrawal.
**4. Attention and cognitive control** with concentration difficulties, distractibility, and low impulse control.
**5. Approach motivation, mechanisms of reward learning and social processes such as social communication, perception and understanding self and others and self knowledge** are valuable salutogenic life skills which should be addressed in treatment of adolescent depression.

Since a clinical application is time-limited and constrained by aspects of feasibility, we propose the following simplification in the translation of the RDoC constructs to a progressive skills-training with clear treatment goals for adolescent depression (see **Table 1**):

1. Improve autonomic regulation and decreased hyper-arousal and limbic hyper-reactivity (with hypothesized secondary effects on sleep disturbances).
2. Practice attention and interoceptive awareness to enhance dynamic switching away from self-evaluative processing and rumination.
3. Promote emotion recognition by training interoceptive awareness and enhance prefrontal cortical regulation of affect by practicing labeling and communication of emotions.
4. Train top-down cognitive control over affective responses such as negatively biased states of attention, processing, thinking, memory, rumination, and dysfunctional attitudes.
5. Identify intrinsic values to drive pro-social behavior and increase motivation for committed behavioral activation.

## PROPOSED THERAPEUTIC TRAINING STRATEGIES BASED ON CURRENT SCIENTIFIC EVIDENCE OF EFFICACY

The first aim of our intervention is to decrease limbic hyper-reactivity and increase autonomic regulation through the promotion of vagal afference. We aim to target neurocircuitry described in the RDoC construct of sustained threat within the domain of negative valence and the dysfunction in the domain of arousal and regulatory systems both within the RDoC constructs of arousal and sleep-wakefulness. [**Section “Module 1: Calming Down and Creating a Sense of Safety (Weeks 1–3)” for description of the corresponding section of the manual.]**

The nervous system is designed to evaluate risk and match neurophysiological state with the actual risk of the environment (McEwen, 2003). Adolescents with emotional dysregulation often perceive the environment as being dangerous or threatening even when it is safe, which could be a result of early life stress exposure as described in section “The Role of Sustained Threat in Relation to Systemic Dysregulation and the Somatic Manifestations of Depression.” This mismatch may result in defensive states, limbic hyperactivation and sympathetic arousal. On the other hand, there is evidence that during the experience of a safe environment, the influence of afferent vagal motor pathways increases, which in turn inhibits the defense mechanisms of the sympathetic nervous system, dampening the stress response (Porges, 2009).

In adult depression (Licht et al., 2008), as well as in adolescent depression, amygdala hyperactivity is related to a decrease of vagal inhibitory control (Yang et al., 2007; Henje Blom et al., 2010) and in depressed patients, a direct association between depression symptom severity and the modulation of cardio-vagal activity has been shown (Agelink et al., 2002). It has been proposed that individual differences in heart rate variability (HRV) predict attentional inhibition, which suggests that successful inhibition as well as novelty search may be mediated by cortical inhibitory mechanisms among people with high cardiac vagal tone (Park et al., 2012). Cardiac vagal tone is also associated with both adaptive top-down and bottom-up modulation of emotional processing, which is implicated in depression (Park et al., 2013). From both animal and human neurochemical and neuroimaging studies as well as from studies using vagus nerve stimulation (VNS) – a therapeutic brain stimulation technique sending electrical impulses to the left cervical vagus nerve, which is approved as an adjunct long-term treatment for chronic or recurrent depression – there is considerable evidence that the vagus nerve and its stimulation influence limbic and higher cortical brain regions implicated in mood disorders (Park et al., 2007; Vonck et al., 2014). RSA-biofeedback and breathing training has also been shown to increase vagal modulation and to reduce depressive symptoms (Siepmann et al., 2008; Patron et al., 2012). Increased HRV, and more specifically, respiratory sinus arrhythmia (RSA), has been shown to relate to the level of engagement of coping strategies and social well-being. Moreover, RSA predicts less use of avoidance strategies for regulating negative emotions and more use of socially adaptive emotion-regulation skills in young adults (Geisler et al., 2013).

A few small studies of respiratory biofeedback with focus on assessing RSA have indicated beneficial effects on anxious and depressive symptoms (Patron et al., 2012; Sutarto et al., 2012). Several studies have shown that regular practice of breathing techniques also substantially changes respiratory metabolism and produces psychological effects, such as reduction of chemo-reflex sensitivity (Spicuzza et al., 2000), oxidative stress (Sharma et al., 2003), depressive symptoms (Tweeddale et al., 1994) and symptoms of panic disorder (Meuret et al., 2005; Wollburg et al., 2011; Kim et al., 2012).

We conclude that breathing exercises may be helpful in increasing vagal afference and improving autonomic regulation and for the purpose of the treatment of adolescent depression, yoga-based breathing and movement practices may improve regulatory skills by recruiting vagal and sensory afferent neurocircuitry. These practices may thus have an effect on limbic hyperactivity, break the negative spiral previously described, and prevent the recurrent course of depression. Despite some methodological limitations, there are some preliminary promising results for yoga-based treatment of depressive symptomatology (Pilkington et al., 2005; Ospina et al., 2008; Uebelacker et al., 2010). Limitations of yoga studies for depression include insufficient description of techniques applied as well as problems with control conditions (Pilkington et al., 2005; Shapiro et al., 2007; Dunn, 2008; Uebelacker et al., 2010; Cabral et al., 2011; Balasubramaniam et al., 2012; Khalsa, 2013). No studies have yet been published on yoga based treatment for adolescent depression but two preliminary RCTs, one in a high-school setting (Noggle et al., 2012) and one in a secondary school setting (Khalsa et al., 2012), have focused on yoga as a school intervention for increased wellness and both suggest preventive effects on mental health. Other strategies to calm a hyperactive limbic system such as sensory activation through sound, light (Canbeyli, 2013), smell (Perry and Perry, 2006; Lv et al., 2013), and touch (Hou et al., 2010) are not within the scope of this review, but may have potential anti-depressive effects that need to be further investigated.

The second priority in our intervention is to increase attention skills and shift neural activity from negative self-referencing processing to present-moment sensory and interoceptive awareness. We aim to target parts of the neural circuitry implicated within the RDoC construct of loss in the domain of negative valence and the construct of attention in the domain of cognitive systems. **[Section “Module 2: Attending and Caring about Our Inner Experience (Weeks 4–6)” for description of the corresponding section of the manual.]**

Adults with MDD who paid less attention to their emotions showed better recovery from MDD (Thompson et al., 2013), but paying too little attention to one’s inner state is also thought to be maladaptive for depressed individuals (Thompson et al., 2013). Attending to emotions may be beneficial for individuals with a good capacity to regulate mood, but may be potentially detrimental to those with a low capacity to regulate mood (Lischetzke and Eid, 2003). Consequently, we suggest that practice of attending emotion is prioritized in the intervention, once regulatory skills have been acquired in section “Module 1: Calming Down and Creating a Sense of Safety (Weeks 1–3).”

The insula may be involved in integrating diffuse feedback from the viscera into cognitive awareness (Nieuwenhuys, 2012). Functional MRI experiments have demonstrated that the insula plays an important role in the experience of pain and the experience of several emotions, including anger, fear, disgust, happiness, and sadness (Craig, 2009). Internal body states are represented in the insula and contribute to our subjective feeling, with insular activity correlating positively with interoceptive accuracy (Critchley, 2005). Depressed adults show less accurate heartbeat perception compared to healthy controls (Furman et al., 2013) and in adults with depressive disorder, training of interoceptive awareness seems to enhance focused attention, which is a cognitive process supported by the ACC and the lateral PFC (Farb et al., 2012). In the context of emotion regulation, increasing interoceptive awareness requires reducing self-evaluative processing. The corresponding neural events during this transition to present-moment sensory awareness include a shifting from midline structures of the PFC to the thalamus, insula, and primary sensory regions (Farb et al., 2012). Training of interoceptive awareness seems to buffer against rumination and negative emotional bias (Farb et al., 2012) and a reduction of emotional limbic hyperactivity has also been shown to be one consequence of mindfulness interventions (Paul et al., 2013). Based on these data, we propose that disengagement from internal self-focus by increasing sensory and introceptive moment-to-moment awareness may be especially helpful for reducing depressive symptoms and vulnerability to depression for adolescents.

As discussed in the previous section, we suggest these skills are cultivated predominantly in contemplative movement practice or in body scans (guided practice of interoceptive awareness) that closely follow a movement practice, rather than in sitting meditation. Four hundred clinical trials on meditation-based interventions for clinical disorders (published between 1956 and 2005) were reviewed in 2007, yet the authors could not make any decisive conclusions with respect to effects on depression due to poor methodological quality (Ospina et al., 2008). In 2007, 15 mindfulness based stress reduction (MBSR) studies for depression and anxiety were also reviewed (Toneatto and Nguyen, 2007). MBSR is 8 week group intervention developed by Kabat-Zinn (1996) containing different components of practice such as body scans, sitting meditation, yoga and informal mindfulness in every day life. Meta-analyses of these studies showed that when active control groups were used, MBSR did not show a reliable effect on depression and anxiety (Toneatto and Nguyen, 2007). More recently, a meta-analyses of mindfulness-based practices for the treatment of adult depression showed relatively small effect sizes on self-assessed depression symptom severity: Cohen’s *d* = 0.30 [95% CI, 0.00–0.59] at 8 weeks and Cohen’s *d* = 0.23 [95% CI, 0.05–0.42] at 3–6 months (Goyal et al., 2014). Preliminary results from studies applying modified versions of mindfulness based cognitive therapy (MBCT) for the treatment of acute depression have also been published (Eisendrath et al., 2011, 2014; Munshi et al., 2013). However, the MBCT model does not contain any practices that stimulate the vagal afference such as breathing or movement. The implicated mechanisms of change in the MBCT intervention are rather top-down regulatory skills training and meta-cognition strategies, which we propose should not the primary focus in treating adolescent depression. A recent study by Williams et al. (2013) showed that MBCT provided significant protection against depressive relapse, but only for participants who experienced childhood trauma, which supports the notion that sustained threat neuro-mechanisms may increase the risk for recurrent episodes. In comparison to an active control condition and to treatment as usual (TAU), MBCT did not improve symptoms in patients with recurrent depression.

Carmody and Baer (2008) investigated the components contained in the MBSR program and the amount of time these components were practiced between sessions in relation to outcome. Specifically, a strong association was found between yoga and improved mindfulness skills, reduced psychological symptoms (such as anxiety and inter-personal sensitivity), and improved well-being, even though the yoga was practiced on fewer days and for fewer total hours than the other formal practices (Carmody and Baer, 2008). From these reviews, we can conclude that mindfulness-based interventions alone have limited efficacy for treating adult depression, but body-based contemplative practices may be beneficial and warrant further examination.

Studies of mindfulness-based interventions for adolescent depression in clinical settings are rare and the possible neural mechanisms involved have not been studied in this population. One RCT showed a decrease in depression and anxiety symptoms after a MBSR course delivered in a group format for adolescents with heterogeneous diagnoses in a clinical outpatient setting (Biegel et al., 2009). A pilot RCT of acceptance and commitment therapy (ACT) for individual treatment of teenage depression demonstrated greater improvement in depressive symptoms as compared to TAU (Hayes et al., 2011). Recent studies of mindfulness-based group interventions in school settings for teenagers and young adults have also yielded reductions in depression and anxiety-related symptoms. In a non-randomized study of secondary school students, Kuyken et al. (2013) showed that relative to children who participated in the usual school curriculum, children who participated in a mindfulness intervention reported significantly fewer depressive symptoms post-treatment and at 3 months follow-up. In a cluster-RCT of a mindfulness group program for an adolescent school-based population (ages 13–20 years), Raes et al. (2013) demonstrated that effects on depressive symptoms from baseline to post-intervention and from baseline to 6 months after treatment ended were small to medium (both Cohen’s *d* > 0.30). These promising early results suggest potential for improvement with contemplative practices carefully tailored for maximum skill uptake and neural effect among adolescents with depression.

The third target is emotion regulation by recognition, labeling and communication of emotions, aiming to improve function in neurocircuits within both the constructs of sustained threat and the construct of loss in the RDoC negative valence domain. **[Section “Module 3: Recognizing, Regulating, and Communicating Emotions (Weeks 7–9)” for description of the corresponding section of the manual.]**

We propose that adolescents need to first achieve bottom-up regulatory skills to calm the limbic system with vagal and sensory afferent techniques prior to engaging effectively in training top-down cognitive control strategies. In the previous modules, participants have practiced breathing exercises, yoga-based movement, and body scans to improve attention, interoceptive awareness, and recognition of emotions and their bodily representations. In this module, we introduce more top-down strategies such as labeling emotions. In traditional mindfulness programs for adults, labeling emotions is an inner process that is recommended during meditation when strong emotions arise. It has been shown in adults that mindfulness is associated with enhanced prefrontal cortical regulation of affect through labeling of negative affective stimuli (Creswell et al., 2007), and that during the labeling of emotions, the VLPFC exhibits a dampening effect on the activity of the amygdala (Lieberman et al., 2007; Torrisi et al., 2013). While emotion recognition and labeling are core practices in most mindfulness programs, the skills of communicating emotion or interpreting emotional states in others are not emphasized. We have addressed this limitation by integrating aspects of psychotherapeutic traditions with some evidence of treatment effect for emotional dysregulation and depressive symptomatology in adolescents such as dialectic behavioral therapy (DBT; Bedics et al., 2013) and ACT (Hayes et al., 2010, 2011).

Once the previous regulatory skills have been acquired, the final intervention goal is to introduce practices of top-down cognitive control over affective responses such as negatively biased states of attention, processing, thinking, memory, rumination and dysfunctional attitudes. These functions are related to altered neural circuitry implied in the construct of cognitive control within the RDoC cognitive systems. We also target mechanisms within the RDoC domain of positive valence related to experiential avoidance such as approach motivation and reward learning and within the domain of social processes. **[Section “Module 4: Core Values, Goal Setting and Committed Action (Weeks 10–12)” for description of the corresponding section of the manual.]**

Cognitive-behavioral strategies for managing depressive symptoms seem to rely on improving the function of the PFC and enhancing the cortico-limbic circuits in modulating emotional processing (Mor and Winquist, 2002; Drevets et al., 2008; Beevers et al., 2010; Cooney et al., 2010; Disner et al., 2011). In the TARA intervention, we emphasize the importance of waiting to introduce this approach once the foundation of the previous “bottom-up” regulatory skills have been consolidated. Our rationale for this is that while strategies of cognitive reappraisal can be effective for emotion regulation in healthy and perhaps depressed adults (Denny and Ochsner, 2013), depressed adolescents have fundamental difficulties with cognitively regulating negative emotion due to developmental and possible depression-related limitations in top-down neural circuitry. Top-down cognitive reappraisal training may be maladaptive and increase risk for rumination and contribute to sustained dysphoric mood (Farb et al., 2012). The top-down model of depression treatment does not take into account that the teenage brain has limited capacity for higher-level executive function. These limitations among adolescents may make them less likely to experience long-term anti-depressive benefit from cognitive control strategies as compared to adults, especially under stressful conditions when limbic hyperactivity tends to override top-down cognitive control. Training adults with depression in executive tasks is associated with increased DLPFC activity during cognitive tasks and with decreased amygdala reactivity in response to emotional stimuli (DeRubeis et al., 2008). However, remission of depression after CBT is associated with decreased, basal DLPFC metabolism, suggesting that recovery might involve the lowering of tonic resting-state activity, allowing for greater reactivity when executive control is recruited (Ressler and Mayberg, 2007; DeRubeis et al., 2008). Since adolescents have limited top-down regulation, we suggest that this mechanism may not be the most effective target to reduce depressive symptomatology. Instead, we prioritize an initial bottom-up regulation strategy to help dampen limbic hyper-reactivity and thereby facilitate the DLPFC contribution to emotion regulation. CBT is the recommended choice of psychological treatment for adolescent depression and has yielded modest effect sizes (Weisz et al., 2006; Watanabe et al., 2007; Reinecke et al., 2009). In a large RCT performed for treatment of adolescent depression, CBT showed a response rate of 43% as compared to 35% for placebo during 12 weeks of treatment, which leaves room for future improvement and development of psychological treatments, such as our approach (Reinecke et al., 2009).

## THE CREATION OF A PROGRESSIVE MODULE-BASED TREATMENT PROGRAM FOR ADOLESCENTS WITH DEPRESSION – TRAINING FOR AWARENESS, RESILIENCE, AND ACTION

To enhance the translation of neuroplasticity and neurocircuit retraining research into an effective clinical intervention for adolescent depression in line with RDoC principles, we have integrated approaches drawn from several different paradigms and traditions based on their efficacy and congruence with our scientific theory of change (Cramer et al., 2011). We developed a 12-week group treatment program: we do not aspire to teach meditation or yoga as spiritual practices according to any specific tradition or lineage. The TARA treatment program is only informed by Eastern practices such as meditation and yoga and current evidence on their therapeutic efficacy. The practices have been taken out of their original spiritual and cultural context and then simplified and adapted to fit our therapeutic model and current scientific paradigms. The TARA model has been influenced by practice modalities found in MBSR and MBCT based on evidence of their efficacy for the relevant domains of function as previously outlined (Desrosiers et al., 2013). Some components of the TARA have also been inspired by approaches from CBT, Behavioral Activation, DBT, ACT, Compassion Therapy, and expressive arts therapy. In our proposed research design we include adolescents who are seeking psychiatric care or are under treatment for depressive problems and randomize them to the TARA intervention and TAU or only TAU. Potential participants are screened for depressive symptoms based on DSM-IV, but we do not require a primary MDD diagnosis for inclusion, only a cut-off score of depressive symptoms based on the Children’s Depression Rating Scale Revised (CDRS-R; Poznanski, 1996; Guo et al., 2006). It may be argued that this way of identifying participants to the study is paradoxically based on the very same DSM criteria that we are questioning instead of on pure dimensionality. We acknowledge this as a potential limitation in our present design, but find no other solution in the clinical environment in which the study is conducted, and at a time-point when validated self-assessment of RDoC constructs adapted for age and gender are not yet developed. The DSM scheme just allows for a major subtype distinction that will help focus the proposed therapy. We exclude only on the basis of restrictions in the ability to attend the group format and the training content, for example learning disability, psychosis, severe behavioral problems, severe posttraumatic stress disorder (PTSD) and active suicidality but aim in general to be as inclusive as possible when it comes to psychiatric comorbidities.

The TARA program is constructed in four modules, which for future clinical applications will allow for flexibility and adaption based on participant needs so that a module can potentially be continued or repeated until skill uptake is consolidated (Weisz et al., 2011). Each of the 12 TARA sessions is 1.5 h in length and designed for an optimal group size of 10–12 participants. Before the program starts, a session is offered for parents/guardians or other adult persons such as relatives or family friends that are important in the young person’s life and who may provide a support system in between sessions. The aim of this introductory session is to outline the overall goals of the program, invite the adults to try some of the practices, and guide them in how they can be of support for the adolescent. Home practice is encouraged and between session treatment support, such as audio-recordings of short, guided meditations, is also provided.

The TARA program is interactively designed and relies on understanding participant behavior given their present context and history (Hayes et al., 2010; Levin et al., 2012). A calm, safe, and respectful group climate is essential and promoted by non-judgmental, empathic, and caring attitudes, and predictability and authenticity on the part of the facilitators. The facilitators must be experienced in leading adolescent group processes and committed to personal contemplative practice in order to effectively model the skills and be able to relate to what the participants are experiencing. The content of the program is transmitted both through implicit and explicit learning. New ways of relating are implicitly taught to the participants by employing skillful and authentic ways of dealing with difficult emotions. Brief psycho-educational modules are offered to provide supportive instruction in lifestyle factors that encourage uptake of the core skills in each module, e.g., physical exercise (Cooney et al., 2013), anti-inflammatory diet/nutrition (Lucas et al., 2013), and adequate sleep (Manglick et al., 2013). In this way, adolescents learn core principles drawn from the theoretical and empirical foundation for the program so they can understand how their brains work and thus develop motivation to practice what they are learning.

The program has been “semi-manualized” to make it replicable and easily implemented in different cultures and settings. TARA is based on the principles of neurocircuitry dysfunction and does not require a strict manualization, as long as these mechanisms of change are deeply understood by the facilitators. This approach increases authenticity and allows for personal teaching strategies, adaptions, and flexibility (e.g., certain practices such as respiratory biofeedback that are consistent with the framework can be incorporated). Each session follows the same sequence: first is “the opening of the circle” in which the group members gather to share experiences from the previous week and go through the homework. Next, there is brief psycho-education, followed by breathing exercises, yoga-based movement and a meditation practice. The sessions end with “the closing of the circle” in which the participants give feedback on the session and are given home practice instructions for the following week. Each session component has a distinct and independent progression throughout the 12 weeks. It is therefore possible to break out each single component into a separate 12-week independent program, which will facilitate future studies of the efficacy of each component such as is possible in adaptive trial designs.

### MODULE 1: CALMING DOWN AND CREATING A SENSE OF SAFETY (WEEKS 1–3)

We propose that the first target in correcting the pathophysiology of adolescent depression is to address pathological limbic activity through afferent vagal pathways. We instruct adolescents in breathing techniques and movement practices drawn from yoga that can impact those afferent vagal pathways. As opposed to practices of attending to the breath and increasing awareness of the breath as is typically done in mindfulness-based interventions, we include active breathing practice of a soft “yogic” breathing technique. Slowing down the breathing rate and extending the length of exhalation increases vagus tone and improves autonomic regulation. Our clinical experience suggests that controlling breathing rate and the duration of the breath in this manner promotes feelings of relaxation and well-being. We recommend that participants practice this simple down-pacing and softening of the breath on a daily basis.

After learning to slow and soften their breathing, participants next receive instruction in the use of the “Ujjayi” breathing technique often used in yoga. Instead of using the Sanskrit term “ujjayi” when describing it to the adolescents, we use the term “ocean breath.” In our experience, adolescents are more receptive to new mind/body practices when the instructor uses developmentally appropriate language. Ocean breath practice in the TARA intervention is presented as a deep slow rhythmic breathing with the instruction to visualize the inhalation starting from the lower belly, continuing in a wave through the ribcage, upper chest and throat, and followed by exhaling in the opposite order. Inhalations and exhalations are usually approximately equal in duration, but for the purpose of calming the mind, this practice involves a slight extension of the exhalation along with a very brief pause at the end of each inhalation and exhalation, but without tension or any holding of the breath. Both inhalation and exhalation are done through the nostrils (versus through the mouth). Concurrently, narrowing of the throat passage at the glottis level creates a soft and soothing sound that can be described as sounding like the ocean. The duration of the breath is controlled by the diaphragm, and gets extended by practice, but should never create tension or distress. Participants are encouraged to be gentle with themselves and not force the breathing. The breath can also be used to direct attention to certain parts of the body, for example, to increase interoceptive or sensatory capability, release tension or extend the range of movement. Ujjayi breathing is often used in synchronization with movement and an advantage to practicing breathing techniques in combination with movement in a group setting is that a generalization to ways of using the breath practices in everyday life situations is facilitated. For example, participants are encouraged to use ocean breath along with their movements while walking between classes in school or in regulating their emotions in everyday situations (see “Stop-Breathe-Act” exercise in Module 3).

Following basic instruction in the breathing practices, we offer a series of movement practices drawn from yoga asanas that are intended to promote calmness and “grounding.” The movements are presented in a non-competitive manner that emphasizes experience versus achievement. We do not expect that the hypothesized antidepressant effect of the practice will arise from the intensity of the physical training such as is highlighted in muscle-building, endurance or aerobic fitness. Instead, we promote specific qualities of the movement practices such as posture, balance, and development of equanimity, and non-straining/non-pushing. We support these attitudes and qualities of physical movement to enhance the development of their psychological counterparts. We also emphasize a focus on interoceptive and sensory awareness, along with ways to use the breath to direct the attention toward unpleasant sensations. When participants feel a painful, unpleasant, or stressful physical sensation, we encourage them to use their breathing practices to turn toward the sensation and explore it instead of avoiding the experience. This strategy can thereby lessen the tension related to “holding” emotional pain, stress, or anxiety. At home, a daily practice is preferred as opposed to more extensive training with longer intervals in between practice sessions. Regular, brief practice may be preferable for impacting change in pathophysiological mechanisms.

The psycho-education part in Module 1 covers stress, breathing physiology, sleep, and a discussion about what really matters in the lives of the participants, i.e., defining one’s core-values.

### MODULE 2: ATTENDING AND CARING ABOUT OUR INNER EXPERIENCE (WEEKS 4–6)

Module 2 focuses on attention regulation and practices of interoceptive awareness. In these practices, participants receive verbal guidance to practice paying attention to a variety of “objects of attention” (i.e., the stimuli to which one directs attention). The first set of attention objects includes sensory stimuli such as externally oriented sounds, smells and tactile sensations. Progressively, the focus of attention is shifted inward toward more subtle sensations. Interoceptive awareness may include attention to different types of somatic perceptions for example proprioceptive (a sense of where your body is in space), kinesthetic (a sense of body position, weight, muscle tension and movement), pain, and temperature sensations. The practice of attention and interoceptive awareness can be integrated with yoga-based movement and can also be extended to involve any kind of physical activity, such as walking, running or biking, so that it fits with the culture and interests of the adolescent. Gradually, brief practices in stillness, such as body scans and guided sitting meditations are introduced. Focusing on the breath is a traditional meditation practice and shifting from manipulation of the breath to simply attending to it may be a next step after the breathing practice introduced in Module 1.

Traditionally, mindfulness-based interventions aim to increase the capacity to experience a full range of emotions and sensations instead of trying to ease or modify the experience. From our clinical experience of teaching mindfulness-based methods to adolescents with depression, this approach is often initially experienced as overwhelming and can be counter-productive for adherence and compliance to training. It is important to introduce attention training in a manner that is engaging, relevant, and manageable for the depressed adolescent in order to empower them and motivate continued practice. We hypothesize this practice of attending to one’s inner sensations may increase the ability to capture early warning signs of stress or depression and prepare the practitioner to become more aware and skillful in handling bodily expressions of emotions, which will be the focus of the next module. Teenagers are often preoccupied with handling peer pressure, are vulnerable to the judgments of others, and seek external reward. Caring for one’s own inner experience may increase the capacity for self-compassion and also facilitate empathy and compassion for others, all of which have been shown to increase resilience to depressive problems (Germer and Neff, 2013; Gilbert, 2014). We suggest that the heavy cultural bias to external stimuli and extrinsic value systems may lead to objectification, loss of internal points of references, and increased vulnerability to depressive illness. Thus, we hypothesize that practices of interoceptive awareness can be used to develop the capability to recognize one’s own intrinsic values, and by practice make them a foundation for behavior and action (see Module 4).

The educational topics in Module 2 include basic brain function and how the TARA practices promote the brain’s capacity to regulate feelings of stress, anxiety and depression. The concept of attention is also explained and how attention can improve with practice. Healthy food and eating habits in relation to glucose regulation and mood are also explained.

### MODULE 3: RECOGNIZING, REGULATING, AND COMMUNICATING EMOTIONS (WEEKS 7–9)

The skills training provided in Modules 1 and 2 are continued throughout this module for consolidation and to encourage the application of these practices as a foundation for emotion regulation. Breathing exercises in which exhalation is prolonged are introduced for calming effects and a simple exercise “Stop-Breathe-Act” is practiced with the aim of being able to use breath for emotion regulation in every day life. In Module 3, the practice of interoceptive awareness extends from being focused on body sensation to emotion recognition. The main focus of this module is development of the ability to link bodily sensations to present-moment emotional awareness. As children and adolescents with mood disorders often have alexithymia tendencies (Gulec et al., 2013), externalization techniques and non-verbal forms of describing emotion are included early in this module. For example, emotions can be depicted through artwork so the emotions can be recognized and described in terms of color, texture, shape, and weight. Labeling of emotions is the next step and it is practiced first as a strategy to regulate emotion and then as later as a way to communicate emotion.

The next goal of this module is to use the acquired skills to communicate emotions to others in an empathic, responsible and pro-social way. Practices of recognizing and handling emotions in other people will be practiced. Toward the end of Module 3 there is also an introduction to understanding social triggers that cause negative emotions to arise and a focus on one’s own experiential avoidance strategies and how they may impede obtaining desired life-goals.

The education portion of Module 3 focuses on explaining a schematic illustration of how the brain functions. To further enhance the participants’ autonomy in their practice and to empower them, they start guiding the breathing, yoga-based movement and brief meditations to each other under supervision of the facilitators.

### MODULE 4: CORE VALUES, GOAL SETTING AND COMMITTED ACTION (WEEKS 10–12)

After basic regulatory skills have been acquired, more conventional cognitive reappraisal techniques to skillfully handle negative emotion regulation are introduced. Habitual thought patterns, assumptions, and cultural bias are tested and perspective shifting is practiced. Participants reassess the previously identified social triggers that cause negative emotions to arise for them and how their own experiential avoidance strategies impact their lives. In this last module, there is also an emphasis on summarizing personal core values. This has been a thread throughout the treatment, but it is now addressed directly through written and experiential activities. Each participant is supported in the process of finding out what they intrinsically value and then extending these values to behavior and action oriented toward future short and long-term life goals. Identifying core values may thus serve as a motivational force for sustainable behavioral activation in a way that is meaningful and relevant to the adolescent.

Since adolescents with depression often have social difficulties there are also specific exercises on how to connect and contribute to others, such as teamwork exercises. We investigate different aspects of care and compassion, i.e., toward oneself and others along with the capacity to be able to receive care and compassion. These concepts are practiced in short, guided loving-kindness meditations and by behavioral practices such as “random acts of kindness.” The concepts of caring and compassion are also linked back to core values (i.e., we care about what we value and what is really important to us). Caring is also addressed in a more extended perspective. We hypothesize that explicit practices of how to care for each other will increase a sense of connectedness and decrease the sense of loneliness and separation that depressed adolescents often feel. Furthermore, we hypothesize based on our clinical experience that depressed adolescents often feel disconnected from their environment and lack a sense of purpose and meaning. Adolescents are sometimes left alone with relevant questions and concerns about an uncertain future and without the tools or the knowledge of how to be able to connect and contribute in meaningful ways. Consequently, we have integrated these topics throughout the TARA treatment by practicing care for one another and the world around us. Thus, in this last module, these issues are more explicitly addressed and concrete ways to engage and commit are identified and explored.

Finally, great care is given to end the TARA program in a skillful way. Facilitators guide discussions centered on the following questions, among others: “What have I learned?” “How do I move forward from here?” “What do I do if I feel worse again?” The participants are encouraged to create their own personal daily practice aligned with their life goals. In this way, the breathing, yoga-based movement, and meditations are personalized to fit each participant and can be used as a foundation for life as they have been the foundation of the TARA intervention.

## SUMMARY

In this conceptual article, we describe the design of a semi-structured, progressive, and individually adaptable program based on the NIMH RDoC criteria for the treatment of adolescent depression without major anhedonia. The program prioritizes the core domains of function hypothesized to drive the pathophysiology of adolescent depression and employs straightforward and feasible treatment strategies that have been selected based on current scientific evidence. A limitation of the RDoC matrix is that the developmental aspects of adolescent depression are not well considered and some of the implied neurocircuitry overlaps between domains and constructs. To maximize time, resources, and feasibility, we therefore suggest specific neural circuits to be targeted in our treatment model rather than the full constructs. We hope that this work may form the basis for a novel and more effective treatment strategy for adolescent depression, as well as open up a discussion on how to take on this challenge. We aim to continue the curriculum development of TARA and to test feasibility and efficacy of this program in clinical populations, as well as study the neuroscientific and systemic regulatory mechanisms of change. Finally, we propose this treatment approach will both effectively treat depression and promote more general long-term health and well-being.

## Conflict of Interest Statement

The authors declare that the research was conducted in the absence of any commercial or financial relationships that could be construed as a potential conflict of interest.
